# Is the GnRH Antagonist Protocol Effective at Preventing OHSS for Potentially High Responders Undergoing IVF/ICSI?

**DOI:** 10.1371/journal.pone.0140286

**Published:** 2015-10-15

**Authors:** Weijie Xing, Haiyan Lin, Yu Li, Dongzi Yang, Wenjun Wang, Qingxue Zhang

**Affiliations:** 1 Center for Reproductive Medicine, The Third Affiliated Hospital of Sun Yat-sen University, Guangzhou, China; 2 Center for Reproductive Medicine, Sun Yat-sen Memorial Hospital of Sun Yat-sen University, Guangzhou, China; University of Crete, GREECE

## Abstract

**Objective:**

To determine if the GnRH antagonist protocol is effective in preventing ovarian hyperstimulation syndrome (OHSS) in potentially high responders.

**Methods:**

A total of 660 IVF-ET/ICSI cycles were retrospectively identified. The inclusion criterion was age ≤ 30 years. Cycles were divided into two groups: a GnRHa group and a GnRHant group. In the GnRHa group, the patients received one single injection of 1.0mg-1.3mg Triptorelin in previous mid-luteal phase. In the GnRHant group, a daily dose of 0.25 mg Cetrotide was initiated when a lead follicle obtained a mean diameter of 14 mm, continued up until the day of hCG administration. The duration of stimulation, total dose of Gn, implantation rate, pregnancy rate, and OHSS rate were compared.

**Results:**

The duration of stimulation, E_2_ level on hCG day, numbers of oocytes retrieved, MII oocytes, and high-quality embryos in the GnRHa group were all significantly more than those in the GnRHant group. In the GnRHa group, 83.53% of cancelled fresh-transferred cycles were cancelled because of high risk of OHSS, which was significantly higher than that in the GnRHant group (43.55%, *P*<0.05). The incidence of OHSS in the GnRHa group was slightly higher than that in the GnRHant group. The implantation and clinical pregnancy rates in the GnRHa group were significantly higher than those in the GnRHant group (37.36% VS 19.25%, 62.78% VS 31.06%; *P*<0.05).

**Conclusions:**

Our study demonstrated that for potentially high responders, the GnRHant protocol can, to some extent, lower the cancellation and incidence rates of OHSS. The GnRHa protocol was superior to the GnRHant protocol in terms of implantation and clinical pregnancy rates.

## Introduction

Ovarian hyperstimulation syndrome (OHSS) is one of most serious complications in assisted reproductive technology (ART). It is a potentially life-threatening condition characterized by ovarian enlargement, pleural effusion, ascites, oliguria, hemoconcentration and thromboembolism [1]. The mortality rate of OHSS is low, with an estimated incidence at 1:400 000–1:500 000, and the incidence of hospitalization is 1.8% [2, 3]. It is difficult to predict OHSS. Several variables have been used for predicting OHSS, including serum E_2_ levels and the number of follicles. However, these diagnostics are still controversial for identifying patients at risk for OHSS [4, 5]).

The GnRH antagonist (GnRHant) protocol, which was introduced in the late 1990s, raised great expectations [6]. Unlike GnRH agonists (GnRHa), GnRH antagonists can cause the immediate and rapid suppression of gonadotrophin production to avoid initial gonadotrophin flare and subsequent pituitary down-regulation [7, 8]. It is simpler for the patient, and the lower total dose of Gn may reduce the burden of infertility treatment, reduce the cancellation rates and improve patient compliance [9]. The procedure was supposed to lower the incidence rates of moderate and severe OHSS and improve ovarian outcomes compared with the GnRH agonist long protocol [10]. Different GnRH antagonist protocols have been discussed in the literature for the past few years. The early literature suggested that the pregnancy rate was decreased in the GnRH antagonist protocol compared with the GnRH agonist protocol [11]. However, recent reports about the GnRH antagonist protocol suggest comparable ART outcomes to those of the GnRH agonist protocol. Although GnRH antagonists have been shown to be safe and effective for women undergoing in vitro fertilization (IVF) and intracytoplasmic sperm injection (ICSI), most clinics currently use them mainly in low responders (e.g., older patients) [12, 13].

There is still no consensus as to which protocol is the best treatment for patients with high risk of OHSS, in terms of their overall incidences of OHSS, drop-out rates, and clinical pregnancy rates. The aim of this study was to determine the effects of GnRH antagonist protocols on potentially high responders undergoing IVF/ICSI.

## Methods

A total of 660 IVF-ET/ICSI cycles from January 2013 to December 2013 were retrospectively identified. The inclusion criterion was age ≤ 30 years. The cycles were divided into two groups: a GnRHa group (437 cycles) and a GnRHant group (223 cycles). This retrospective study was approved by the Third Affiliated Hospital of Sun Yat-Sen University Reproductive Medicine Ethic Committee. The patient records/information was anonymized and de-identified prior to analysis.

In the GnRHa group, the long GnRH-a protocol was the same as previously described [14]. At the mid-luteal phase of the preceding cycle, the patients underwent down-regulation with 1.0 mg-1.3 mg of Triptorelin (3.75 mg Gonapeptyl; Ferring). Recombinant FSH (Gonal-F, Serono, Switzerland) and/or hMG (LiZhu, China) were used at dosages ranging between 37.5 IU/day and 300 IU/day after the complete pituitary suppression was confirmed. The dosages of FSH and hMG were adjusted according to ovarian response. Recombinant hCG (Serono, Switzerland) was given to trigger follicle maturation when at least two follicles reached a mean diameter of 18 mm. Oocytes retrieval was carried out 34–36 hours laterby transvaginal ultrasound-guided puncture of follicles.

In the GnRHant group, ovarian stimulation commenced with 75 IU-300 IU rFSH from day 3 of the menstrual cycle. The dosage of FSH and hMG was adjusted according to the ovarian response, which was assessed by ultrasound and serum E_2_ levels. A daily dose of 0.25 mg GnRHant (Cetrotide, Serono, Switzerland) was initiated when a lead follicle reached a mean diameter of 14 mm and continued until the day of hCG administration. Oocyte retrieval was performed transvaginally 34–36 hours after hCG injection.

The SPSS statistical software package (version 11.0) was used for statistical analysis. Values are expressed as the mean ± SD. The unpaired Student t-test was used to compare means from two groups. The χ^2^-test was used to compare categorical variables. *P* < 0.05 was considered statistically significant.

## Results

### Patient Characteristics and Stimulation Outcomes

A total of 660 IVF-ET/ICSI cycles were retrospectively studied. The patient demographic variables are compared in Table 1. The two groups were similar in terms of body mass index, basal FSH level, proportion of primary infertility and IVF cycles. The duration of stimulation in the GnRHa group was 12.37 ± 2.41 days, which was significantly longer than that in the GnRHant group (10.29 ± 3.78, *P*<0.05). There was a significant difference in E_2_ levels on the hCG day between the two groups (3705.79 ± 1460.49 VS 3056.70 ± 1572.75, *P*<0.05). However, the total dose of Gn was similar between the two groups (2188.05 ± 1053.82 VS 2206.01 ± 1354.14, *P* = 0.851).

**Table 1:** Epidemiologic and stimulation characteristics.

Variable	GnRHa (N = 437)	GnRHant (N = 223)	*P* value
Body Mass Index (kg/m^2^)	20.69±2.54	20.65±2.00	NS
Primary infertility (n, %)	251/437 (57.44%)	133/223 (57.08%)	NS
Duration of infertility (years)	3.57±2.14	5.50±3.81	<0.05
No. of IVF cycles (n, %)	270/437 (61.78%)	148/223 (66.37%)	NS
Duration of stimulation (days)	12.37±2.41	10.29±3.78	<0.05
Total dose of Gn administered (IU)	2188.05±1053.82	2206.01±1354.14	NS
E_2_ level on hCG trigger day (pg/mL)	3705.79±1460.49	3056.70±1572.75	<0.05
Endometrial thicknes on hCG trigger day (mm)	11.87±2.65	10.28±2.34	<0.05

Note: NS = not statistically significant. Values presented as mean ± SD unless otherwise specified.

### IVF/ICSI Outcomes

As shown in Table 2, the numbers of oocytes retrieved, MII oocytes and high-quality embryos in GnRHa group were 16.21±7.58, 13.73±6.67, and 2.80±2.95, respectively, which were significantly more than in the GnRHant group (11.15±8.65, 9.04±6.97, and 2.04±2.40, respectively; *P*<0.05). In the GnRHa group, the number of cancelled freshly transferred cycles was 85 cycles, and 83.53% of them were cancelled because of high risk of OHSS, which was significantly higher than that in the GnRHant group (27/62, 43.55%, *P*<0.05). The incidence of OHSS in the GnRHa group was slightly higher than that in the GnRHant group (2.97% VS 1.79%, *P* = 0.445).

**Table 2:** IVF/ICSI Outcomes.

Variable	GnRHa (N = 437)	GnRHant (N = 223)	*P* value
No. of oocytes retrieved	16.21±7.58	11.15±8.65	<0.05
No. of MII oocytes	13.73±6.67	9.04±6.97	<0.05
No. of high-quality embryos	2.80±2.95	2.04±2.40	<0.05
No. of cancelled fresh transferred cycle (n, %)	85/437 (19.45%)	62/223 (27.80%)	<0.05
No. of cancelled fresh transferred cycle because of high risk of OHSS (n, %)	71/85 (83.53%)	27/62 (43.55%)	<0.05
No. of OHSS (moderate and severe; n, %)	13/437 (2.97%)	4/223 (1.79%)	NS
Normal fertilization rate (%)	66.35%	62.14%	<0.05
Implantation rate (%)	37.36%	19.25%	<0.05
Clinical pregnancy/fresh transferred cycle (n, %)	221/352 (62.78%)	50/161 (31.06%)	<0.05
Miscarriage/fresh transferred cycle (n, %)	8/221 (3.62%)	6/50 (12.00%)	<0.05

Note: NS = not statistically significant. Values presented as mean ± SD unless otherwise specified.

When the χ^2^-test was performed, we obtained the following results: (1) The normal fertilization rate of the GnRHa group was 66.35%, which was significantly higher than that in the GnRHant group (62.14%, *P*<0.05); (2) The implantation rate of the GnRHa group was 37.36%, which was significantly higher than that in the GnRHant group (19.25%, *P*<0.05); (3)the clinical pregnancy rate of the GnRHa group was significantly higher than that of the GnRHant group (62.78% VS 31.06%, *P*<0.05); (4) There was significant difference in miscarriage rate between the two groups (3.62% VS 12.00%, *P*<0.05).

## Discussion

Young patients with good ovarian reserves are at high risk of OHSS. For these patients, selecting an effective protocol is still a frustrating challenge in IVF/ICSI cycles. The most optimal protocol for high responders should have an acceptable rate of cancellation, obtain moderate healthy mature oocytes and high quality embryos at a reasonable cost and duration of therapy, provide a suitable endometrium for implantation, and have maximal pregnancy and live birth rates [15]. For over 20 years, GnRH agonists have been used to prevent the luteinizing hormone (LH) surge that results from multiple follicular development [1]. However, more and more studies are demonstrating that the GnRH agonist protocol can induce severe OHSS, especially for potentially high responders.

The GnRH antagonist protocol is supposed to reduce OHSS. Many researchers have demonstrated that there are several advantages in antagonist methods, including a shorter duration of Gn and a smaller dose of Gn per cycle [16]. Conflicting evidence still exists regarding the superiority of one protocol over the other [17]. In our study, we compared the GnRH agonist and GnRH antagonist protocols in patients with age ≤ 30 years. Our results showed that the duration of stimulation in the GnRHa group was 12.37 ± 2.41 days, which was significantly longer than that in the GnRHant group (10.29 ± 3.78, *P*<0.05). However, the numbers of oocytes retrieved, MII oocytes and high-quality embryos in the GnRHa group were all more than those in the GnRHant group (*P*<0.05). These findings were in agreement with previous studies.

There is still controversy over whether the GnRHant protocol can reduce the incidence of OHSS. A large body of published data shows that the GnRHant protocol can reduce the incidence of mild and moderate OHSS, compared with the GnRHa protocol [18]. However, one meta-analysis including five randomized controlled studies suggested that the incidence of severe OHSS was not associated with the type of analogue [19]. In the present study, we found that in the GnRHa group, 83.53% of cancelled freshly transferred cycles were cancelled because of a high risk of OHSS, which was significantly higher than that in the GnRHant group (43.55%, *P*<0.05). The incidence of moderate and severe OHSS in the GnRHa group was slightly higher than that in the GnRHant group (2.97% VS 1.79%, *P* = 0.445). These results demonstrated that the GnRHant protocol can, to some extent, lower the cancellation rates and the incidence of OHSS.

However, many studies have also demonstrated that pregnancy rates are decreased in the GnRH antagonist protocol compared with the GnRH agonist protocol [11]. This is a key factor explaining why the GnRH antagonist protocol is only used in older patients or patients who have failed in previous IVF/ICSI cycles [12]. Our data from young patients with an age ≤ 30 years showed that the normal fertilization rate, implantation and clinical pregnancy rates in the GnRHa group were significantly higher than those in the GnRHant group (66.35% VS 62.14%, 37.36% VS 19.25%, 62.78% VS 31.06%, respectively; *P*<0.05). The long GnRH agonist protocol is still considered a classical, gold standard protocol for young patients, even though there is a high risk of OHSS [20]. Several reasons can explain why the long protocol was better than GnRH antagonist protocol: (1) The long protocol can lead to complete suppression and lower serum LH level. Under these conditions, the endometrium may be more suitable for embryo implantation. Previous studies have showed that LH may exert direct effects on the endometrium [21]. Some recent studies suggested that lower serum LH levels in the long-acting GnRHa protocol might play a beneficial role for endometrial receptivity. (2) The long protocol, starting in the mid-luteal phase, can obtain a better follicular synchronization, and lead to an increase in size of the follicle cohort recruited for the cycle. So, it can promote better follicle recruitment and more oocytes harvested. In our previous study, the two GnRH-a protocols (long GnRH-a protocol and short GnRH-a protocol) for ovarian stimulation in IVF/ICSI cycles were compared, our results demonstrated that regardless of patient age, the long protocol was superior to the short protocol in terms of number of retrieved oocytes and implantation and pregnancy rates [14]. In this study, our results also showed that the long GnRH-a protocol was superior to the GnRHant protocol. Long GnRH-a protocol is a classical protocol for the patients and has gained widespread popularity.

## Conclusions

In conclusion, our study demonstrated that for potentially high responders, the GnRHant protocol can, to some extent, lower the cancellation rate and the incidence of OHSS. However, the GnRHa protocol was superior to the GnRHant protocol in terms of normal fertilization rate, implantation rates and clinical pregnancy rates. The total dose of Gn should be reduced to prevent OHSS in the GnRHa protocol.
